# Favorable Neurological Outcome Following Prolonged Super‐Refractory Status Epilepticus in a Resource‐Limited Setting: A Case Report

**DOI:** 10.1002/ccr3.73544

**Published:** 2026-09-16

**Authors:** Amanda Sang, Allana Yearwood, Keevan Singh, Vaughn Lalla, Kamille Abdool

**Affiliations:** ^1^ Department of Anaesthetics and Intensive Care San Fernando General Hospital San Fernando Trinidad and Tobago; ^2^ Department of Internal Medicine San Fernando General Hospital San Fernando Trinidad and Tobago; ^3^ Department of Internal Medicine (Neurology) San Fernando General Hospital San Fernando Trinidad and Tobago

**Keywords:** autoimmune encephalitis, electroencephalography (EEG), magnetic resonance imaging (MRI), super‐refractory status epilepticus (SRSE)

## Abstract

Super‐refractory status epilepticus (SRSE) is associated with high mortality and poor neurological outcomes. This case highlights a young male treated for SRSE. Diagnosis and treatment were expansive. Despite the severity of the patient's disease course, the patient had a favorable neurological outcome at the end of his hospital stay.

## Key Clinical Message

Super‐refractory status epilepticus (SRSE) is a neurological emergency associated with high mortality and poor neurological consequences. However, with extensive proper management, a good neurological outcome can be achieved.

## 1 Introduction

Super‐refractory status epilepticus (SRSE) is defined as clinically or electrographically identified seizure activity that persists for more than 24 h despite the initiation of anesthetic therapy, or that recurs upon reduction or withdrawal of anesthetic agents [1, 2]. Approximately 4%–13% of patients with status epilepticus (SE) progress to SRSE [3, 4, 5].

SRSE represents a rare but life‐threatening neurological emergency, with outcomes largely dependent on underlying etiology and the timeliness of therapeutic intervention. However, the underlying etiology remains unknown in up to 22% of cases [6]. To aid early risk stratification, several clinical scoring systems have been developed, including the Status Epilepticus Severity Score (STESS) and the END‐IT score [2], which incorporate clinical and radiological variables to predict mortality and functional outcomes in SE.

Despite the range of therapeutic interventions available, prognosis remains poor for SRSE, with reported long‐term mortality rates ranging from 30% to 50% [2]. A retrospective observational study identified several factors associated with increased mortality, including generalized convulsive status epilepticus, failure of anesthetic tapering, prolonged duration and use of multiple anesthetic agents, and the development of systemic complications such as metabolic and cardiovascular instability [5].

Of those that do survive, it is estimated that only 35% recover to baseline [7]. Among those discharged after SRSE, Cornwall et al. [6] found that 73% of patients had a modified Rankin score ≥ 3, indicating moderate to severe disability. However, the available literature does not distinguish whether this disability is primarily attributable to the underlying neurological disease, seizure‐induced brain injury, prolonged antiseizure therapy, or complications of prolonged intensive care [6]. The Modified Rankin Scale (mRS) is the most used measure of functional outcome after neurological injury ranging from 0 to 6, with higher scores indicating greater disability. Despite adverse prognostic indicators, outcomes in SRSE remain heterogeneous, and favorable neurological recovery may still occur in selected patients.

## 2 Case History/Examination

A 16‐year‐old previously well West Indian male, with no known medical history, no history of alcohol or recreational drug use, and no family history of epilepsy, presented with a reduced Glasgow Coma Scale (GCS) score of 12 following a 5‐day prodrome of coryzal symptoms, intermittent fever, periorbital oedema, lethargy, reduced oral intake, visual hallucinations, and photophobia. Shortly after presentation, he developed generalized convulsive status epilepticus requiring endotracheal intubation and mechanical ventilation for airway protection and seizure control. He was subsequently admitted to the intensive care unit (ICU).

## 3 Differential Diagnosis, Investigations and Treatment

Initial laboratory investigations demonstrated a normal complete blood count and renal function. Liver function tests revealed transaminitis, with an aspartate aminotransferase (AST) of 308 U/L (reference range 10–40 U/L) and alanine aminotransferase (ALT) of 275 U/L (reference range 7–56 U/L). The erythrocyte sedimentation rate (ESR) was elevated with a value of 87 mm/h (reference range < 15 mm/h), whereas C‐reactive protein (CRP) remained within normal limits at 0.027 mg/dL (reference range < 0.5 mg/dL). Autoimmune screening included ANA/ANF, anti‐GBM antibodies, c‐ANCA, and p‐ANCA. ANA/ANF and anti‐GBM antibodies were both negative. C‐ANCA analysis by Western blot was weakly positive at a level of 17 (reference positive range: 11–50), whereas p‐ANCA was borderline at a level of 7 (reference borderline range: 6–10). In the absence of clinical or laboratory features suggestive of ANCA‐associated vasculitis, these findings were considered of little significance. A specific neuronal autoimmune encephalitis antibody panel was not performed due to unavailability of tests. Septic workup, including blood and sputum cultures, with toxicology screening, were negative. Contrast and non‐contrast computed tomography (CT) of the brain demonstrated no acute intracranial abnormality.

On initial presentation, encephalitis was suspected and empirical antimicrobial therapy was commenced. Ceftriaxone 2 g was given intravenously every 12 h to provide central nervous system penetration along with acyclovir 500 mg (10 mg/kg) every 8 h.

On Day 1, the patient received a loading dose of intravenous phenytoin (1 g) followed by maintenance dosing of 100 mg three times daily. Persistent seizure activity, both facial twitching and generalized tonic clonic seizures, necessitated induction of anesthetic agents. This was done using propofol infusion at up to 60 mcg/kg/min and midazolam infusion starting at 0.2 mg/kg/h; however, without access to continuous electroencephalography (EEG) monitoring. Treatment was instead guided by clinical examination and seizure activity. Simultaneously, a noradrenaline infusion was commenced for cardiovascular support. Despite elevated doses of anesthetic agents, persistent facial twitching and intermittent tonic clonic seizures occurred, indicating ongoing refractory seizure activity with limited clinical improvement. Thus, sodium valproate was added on Day 2, followed by phenobarbital and levetiracetam on Day 5 with limited improvement.

Diagnosis was delayed by the absence of in‐hospital electroencephalography (EEG) services and delayed consent for lumbar puncture. Lumbar puncture was ultimately performed on Day 12 and revealed protein of 18 mg/dL (reference range 15–45 mg/dL), glucose of 78 mg/dL, and a white blood cell count of 6.6 cells/μL. Cerebrospinal fluid polymerase chain reaction testing was negative for infectious pathogens.

Despite escalation of therapy, the patient progressed to super‐refractory status epilepticus, with persistent focal and generalized seizure activity throughout a prolonged ICU course. The patient received approximately 15 days of combined propofol and midazolam infusion therapy. These doses were titrated upward with the intent of achieving burst suppression doses. However, continuous EEG monitoring was unavailable; therefore, achievement and maintenance of burst suppression could not be objectively confirmed. Treatment was consequently guided by clinical seizure activity. Additional therapeutic interventions were also added. On Day 12, ketamine infusion was started at 2 mg/kg/h (infusion up to 13 days) along with magnesium sulfate therapy targeting serum concentrations between 3.5 and 5.0 mmol/L (infusion up to 3 days). On Day 13, a ketogenic diet was also implemented. Over a 2‐week period, multiple attempts to wean the anesthetic infusions resulted in recurrent seizure activity, necessitating re‐escalation of therapy.

Given the clinical presentation and exclusion of alternative causes, autoimmune encephalitis was strongly suspected, though neuronal testing was unavailable for confirmation. Empirical immunotherapy with intravenous methylprednisolone (1 g daily for 3 days) and intravenous immunoglobulin (IVIG) at 0.4 g/kg/day commenced on Day 13 and continued for 5 days. Even this had little effect on the observed seizure activity.

The prolonged ICU course was further complicated by a ventilator‐associated pneumonia caused by multidrug‐resistant *Klebsiella pneumoniae* , requiring escalation of antimicrobial therapy according to culture sensitivities. Progressive neuromuscular weakness, severe cachexia secondary to prolonged critical illness, and prolonged ventilator dependence necessitated enteral nutritional support via nasogastric feeding. Owing to persistent neurological impairment, repeated failed attempts at liberation from mechanical ventilation, and the anticipated need for prolonged airway protection, a surgical tracheostomy was performed on Day 16 of ICU admission.

Although the patient remained warded within the ICU, regular multidisciplinary family meetings were conducted involving the intensive care and neurology teams. Relatives were counseled on multiple occasions regarding the guarded prognosis and the high likelihood of significant neurological disability or death given the prolonged course of SRSE. Despite these discussions, aggressive treatment was continued because of the patient's young age and the possibility of a reversible autoimmune etiology.

**FIGURE 1:**
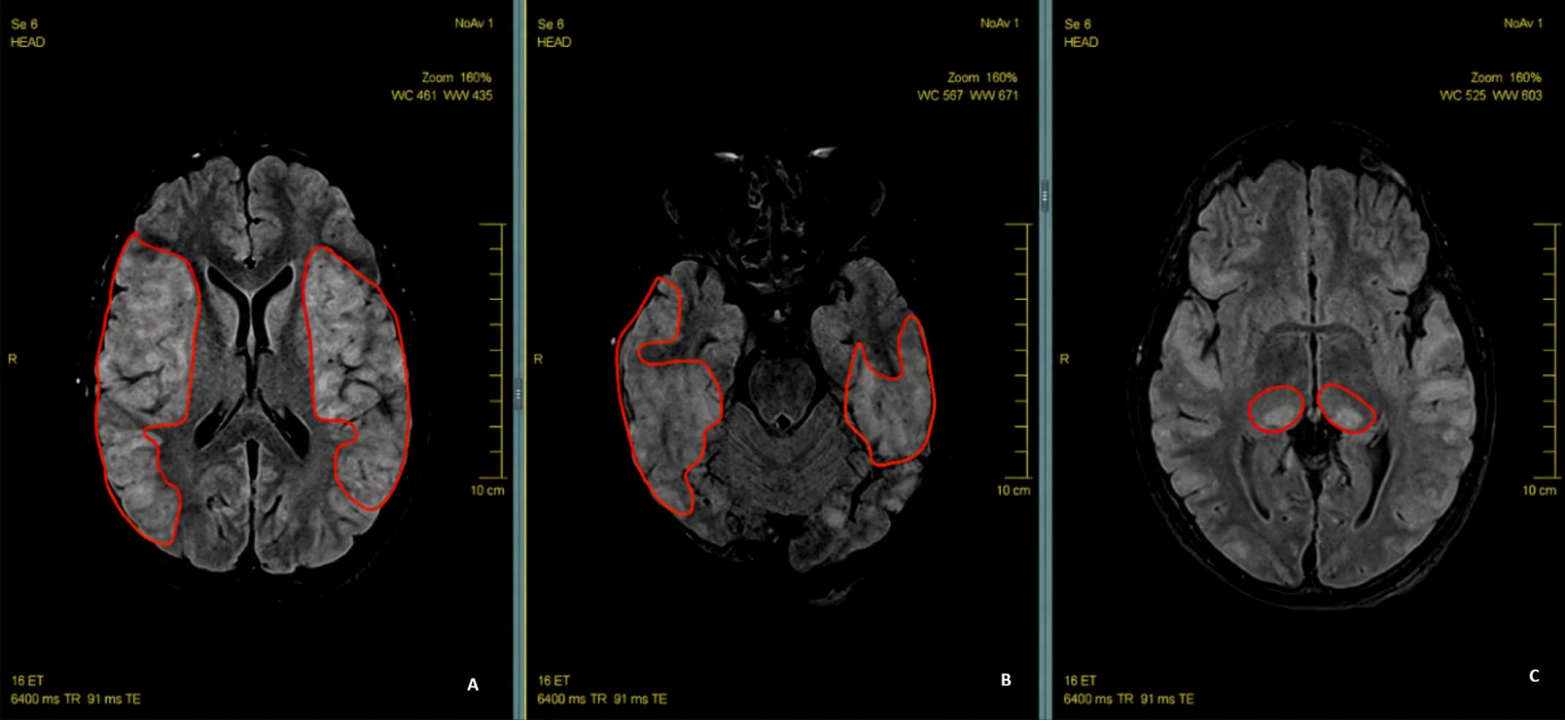
Axial T2/FLAIR MRI sequence demonstrating bilateral temporal and thalamic hyperintensities consistent with autoimmune encephalitis. (A) Bilateral cortical and subcortical temporal lobe hyperintensities. (B) Predominant Bilateral temporal lobe involvement. (C) Bilateral thalamic hyperintensities.

Magnetic resonance imaging (MRI) of the brain was delayed due to clinical instability and was eventually performed on Day 20. MRI demonstrated diffuse bilateral patchy cortical and subcortical T2/FLAIR hyperintense signal abnormalities predominantly involving the temporal lobes and thalami adjacent to the posterior ventricles. Mild diffusion restriction was present without significant contrast enhancement. These findings were considered highly suggestive of autoimmune encephalitis (Figure 1).

Despite ongoing therapy, seizure activity persisted, and lacosamide was added on Day 22. Due to the lack of availability of plasmapheresis at the treating institution, the patient required transfer to another tertiary facility on Day 34 for plasma exchange therapy. Prior to transfer for plasmapheresis, the patient was breathing spontaneously via his tracheostomy with supplemental oxygen therapy and no longer required ventilatory support. Following 4 sessions of plasmapheresis, the patient demonstrated a progressive reduction in seizure frequency with gradual improvement in neurological responsiveness and awareness. He was successfully decannulated prior to hospital discharge. As his neurological function improved, he progressed from nasogastric enteral feeding to full oral nutrition with restoration of adequate swallowing function. Intensive multidisciplinary rehabilitation contributed to progressive improvement in mobility, muscle strength, and functional independence.

After a 32‐day intensive care unit stay and a total hospital stay of 98 days, the patient was discharged home. The patient was discharged on oral sodium valproate, phenytoin, levetiracetam, and lacosamide. At 1‐month follow‐up, he had a Glasgow Coma Scale (GCS) score of 15 and a modified Rankin Scale (mRS) score of 3, indicating moderate disability but functional independence. He was independently ambulant, tolerating a normal oral diet, communicating near his pre‐morbid baseline, and had no further episodes of status epilepticus. Follow‐up outpatient electroencephalography (EEG) demonstrated frequent right frontal epileptiform discharges with occasional generalized spike‐and‐wave activity on a preserved symmetrical background, without electrographic seizures or status epilepticus, consistent with resolution of the acute ictal state and persistence of a right frontal epileptogenic focus.

## 4 Discussion

Super‐refractory status epilepticus (SRSE) represents one of the most severe neurological emergencies encountered in critical care medicine and is associated with both high mortality and long‐term neurological morbidity [2]. Longer ICU courses and mechanical ventilation are necessarily associated with more complications [8]. The median length of stay for patients with SRSE ranged in the literature between 16.5 and 37 days, with higher healthcare costs and higher discharge mortality rates in comparison to all SE patients [4, 9].

When compared to the findings by Fang et al. [5], this case had many of the identified factors for an unfavorable outcome. These included generalized convulsive status epilepticus, failure of anesthetic tapering, prolonged duration and course, use of multiple anesthetic agents, and the development of cardiovascular instability [5]. However, application of prognostic scoring systems yielded discordance with actual clinical outcomes. Both the STESS score of 3 out of 6 and the END‐IT score of 5 suggested higher predicted mortality and a poorer prognosis. This illustrates that prognostic models should complement rather than replace clinical judgment, particularly in young patients with potentially reversible aetiologies.

Several factors may have contributed to the favorable outcome observed. First, the patient was young with no significant premorbid comorbidities. Younger age has been associated with favorable outcomes [6]. One study found that mortality was highest in patients aged over 75 years [10]. Second, the underlying etiology was likely autoimmune encephalitis, a potentially reversible cause of SRSE, when recognized and treated with immunomodulatory therapy. A retrospective observational study of patients with autoimmune encephalitis in comparison to other causes of new onset refractory status epilepticus had better long‐term outcomes [11]. Mortality in SRSE was highest from acute brain injury such as stroke, anoxia, or infection [2].

Prolonged SRSE poses significant critical care challenges that frequently contribute to patient morbidity and mortality. Deep sedation, prolonged mechanical ventilation, hospital‐acquired infections, ICU‐associated weakness with cachexia, and the need for lengthy rehabilitation often become the dominant determinants of outcome [12]. Our patient developed many recognized ICU complications, including ventilator‐associated pneumonia caused by multidrug‐resistant *K. pneumoniae* , prolonged ventilator dependence requiring tracheostomy, severe cachexia requiring enteral nutritional support, and ICU‐acquired weakness necessitating extensive rehabilitation. Successful recovery therefore reflected not only seizure control but also comprehensive ICU management aimed at preventing and managing complications of prolonged critical illness.

Importantly, this case demonstrates that prolonged SRSE should not necessarily be viewed as futile, even in resource‐limited environments. Meaningful neurological recovery may still be achieved despite prolonged seizure duration, significant critical illness complications, and delayed access to advanced therapies. Aggressive multidisciplinary management and persistence with diagnostic and therapeutic interventions remain essential, particularly in young patients with potentially reversible causes of SRSE.

## 5 Conclusion and Results

This case demonstrates that favorable neurological recovery can occur despite prolonged SRSE, extensive critical illness complications, and substantial resource limitations. Young age, a potentially reversible autoimmune etiology, and persistence with aggressive multidisciplinary management may contribute to recovery even when conventional prognostic indicators suggest a poor outcome. Recognition of this possibility may help guide treatment decisions and avoid premature therapeutic nihilism in similar patients.

## Author Contributions

**Amanda Sang:** investigation, supervision, writing – original draft, writing – review and editing. **Allana Yearwood:** data curation, writing – original draft, writing – review and editing. **Keevan Singh:** supervision, conceptualization, writing – review and editing. **Vaughn Lalla:** supervision, resources. **Kamille Abdool:** supervision, resources.

## Funding

The authors have nothing to report.

## Consent

Written informed consent was obtained for the patient, who is a minor, from the patient's parent for publication of this case report in accordance with the journal's patient consent policy.

## Conflicts of Interest

The authors declare no conflicts of interest.

## Data Availability

The data collected in the preparation of this case report is readily available from the corresponding author upon reasonable request.
